# Incidental diagnosis of intestinal perforation on a ^99m^Tc DTPA renogram

**DOI:** 10.22038/AOJNMB.2024.76465.1538

**Published:** 2024

**Authors:** Awiral Saxena, Manjit Sarma, P. Shanmuga Sundaram, Padma Subramanyam, Anwin Joseph Kavanal

**Affiliations:** Department of Nuclear Medicine & Molecular Imaging, Amrita Institute of Medical Science, Kochi, Kerala, India

## Abstract

Perforation of the bowel can be a life-threatening condition and is usually clinically diagnosed when a patient presents with such features as severe abdominal pain, tenderness, and tachycardia. Bowel perforation may be corroborated by various conventional imaging modalities, including X-ray, ultrasonography, computed tomography, and magnetic resonance imaging. Nuclear medicine imaging modalities seldom have a role to play in these settings. Rarely diagnosis of perforation may be missed if it is concealed and does not present with the usual signs. Mostly the perforation will eventually be diagnosed if they develop signs and symptoms and is taken up for an exploratory laparotomy. A delay in diagnosis can later lead to significant patient morbidity or even mortality. This report describes a case where possible intestinal perforation was suspected on a ^99m^Tc-DTPA renogram in a postoperative patient with significant urine leak, the presence of which was confirmed intraoperatively. To our knowledge, this was the first such case in the literature.

## Introduction

 Tc-99m diethylenetriamine pentaacetate (DTPA) is an appropriate method for the relative renal function evaluation (1) and can simultaneously measure glomerular filtration rate (GFR). Compared to other methods, DTPA is relatively simple to perform and avoids the additional expense and technical difficulties for GFR estimation (2). Iatrogenic injury to the urinary tract, including the kidneys, ureters, bladder, and urethra, is a potential complication of surgical procedures performed in or around the retroperitoneal abdominal space or pelvis (3). The incidence of urine leak after partial nephrectomy is approximately 2% (4). 

 Depending on the severity of the injury and the compressive forces, electrolyte imbalances and progressive increases in serum creatinine are laboratory values that can guide the treatment and how emergently intervention needs to be performed (5). Urinomas are often small at presentation and will reabsorb without intervention in most instances. In cases of a more significant injury, fever, urosepsis, or a larger urinoma that is expanding, compressing, or failing to reabsorb, an intervention may be necessitated by urology or interventional radiology to relieve the urinoma (6). This is a case with an atypical presentation where a post-surgical intervention urinary leak was known but no intestinal perforation was suspected.

## Case report

 The patient was a known case of carcinoma colon status post hemicolectomy and right partial nephrectomy (Gerota’s fascia was involved), who had a significant urine leak that did not resolve on conservative management. The patient had multiple episodes of fever with high drain creatinine levels and elevated white blood cell counts, in the post-operative period. 

 A contrast-enhanced computed tomography (CT) was conducted which showed significant urinary leakage from injury to the right proximal ureter near the pelviureteric junction and the collection of contrast in the right pararenal and paraduodenal spaces. Initially, the patient was managed by placing a percutaneous nephrostomy stent. However, the patient’s symptoms did not resolve, and drain creatinine levels were rising persistently, probably requiring an intervention to manage the leak. In planning for a complete nephrectomy, the patient was sent for a ^99m^Tc-DTPA scan for evaluation of the differential function of the kidneys. 4 mCi of ^99m^Tc-DTPA was injected as an intravenous bolus, and initial dynamic images (20 min) and serial static images (immediate prevoid, post-void, and 1 hour delayed images) were acquired using a dual head variable angle Gamma camera (GE Healthcare Optima NM/CT 640). Single-photon emission computed tomography (SPECT)/CT of the abdomen was also acquired at 1 hour post-DTPA injection. 

**Figure 1:**
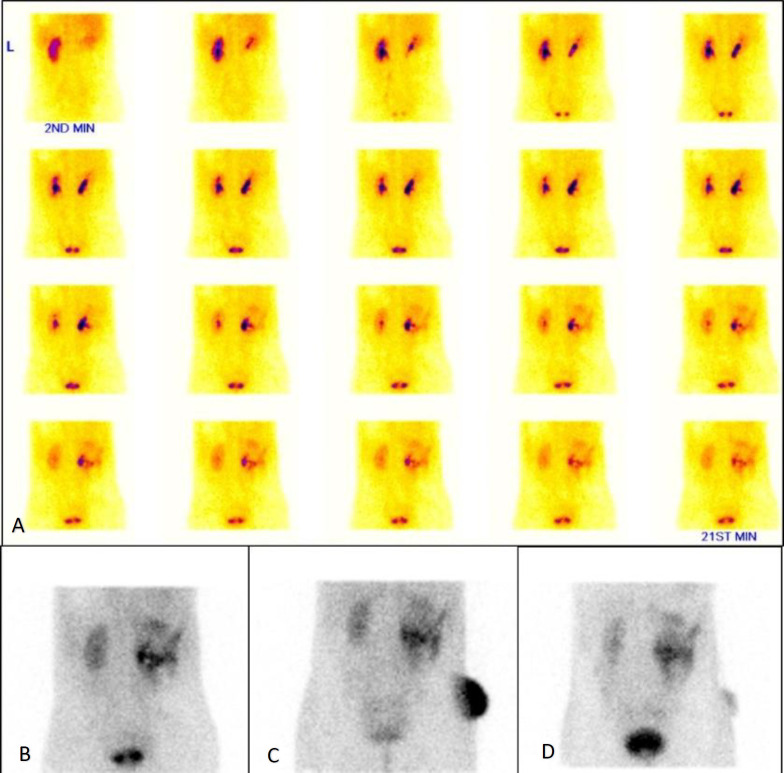
(**A**) Dynamic 1-minute images, (**B**) immediate static prevoid, (**C**) postvoid, and (**D**) 1-hour delayed images showing tracer accumulation in the kidney and leakage of tracer into perirenal and subsequently into the peritoneal spaces. The nephrostomy drainage bag was seen filled on the right side of the patient (**C**) which clears in the delayed image (**D**)

 During dynamic phase acquisition and in static images (Figure 1), tracer accumulation was observed in the kidney, and progressive spillover of the tracer was noted into the perirenal and peritoneal spaces. To know the exact extent of tracer collection in the peritoneal/retroperitoneal spaces, a SPECT/CT of the abdomen was acquired subsequently. 

**Figure 2:**
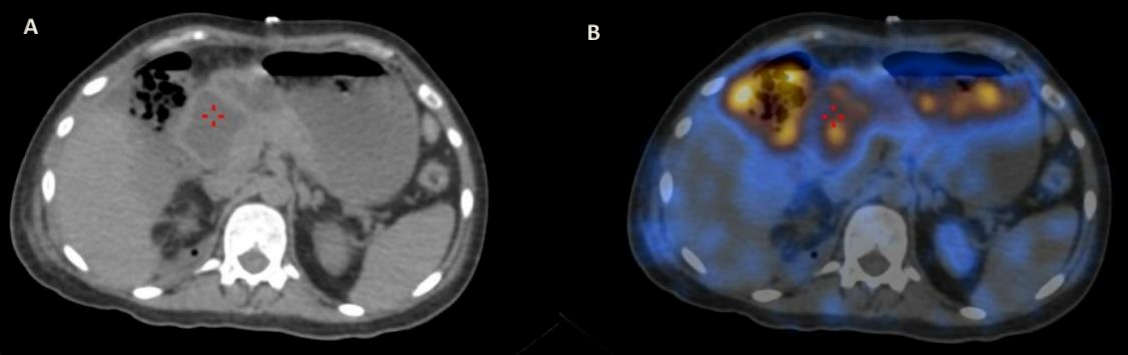
(**A**) Non contrast CT and (**B**) Fused SPECT CT images showing the collection of tracer in the perirenal space, paracolic gutter, and perihepatic spaces

 SPECT/CT images (Figure 2) showed the collection of tracers in the perirenal space, paracolic gutter, and perihepatic spaces. The images also showed significant tracer collection in the bowel loops and also in the stomach. This raised the suspicion of a communication tract between the collections and the intestinal loops. 

**Figure 3:**
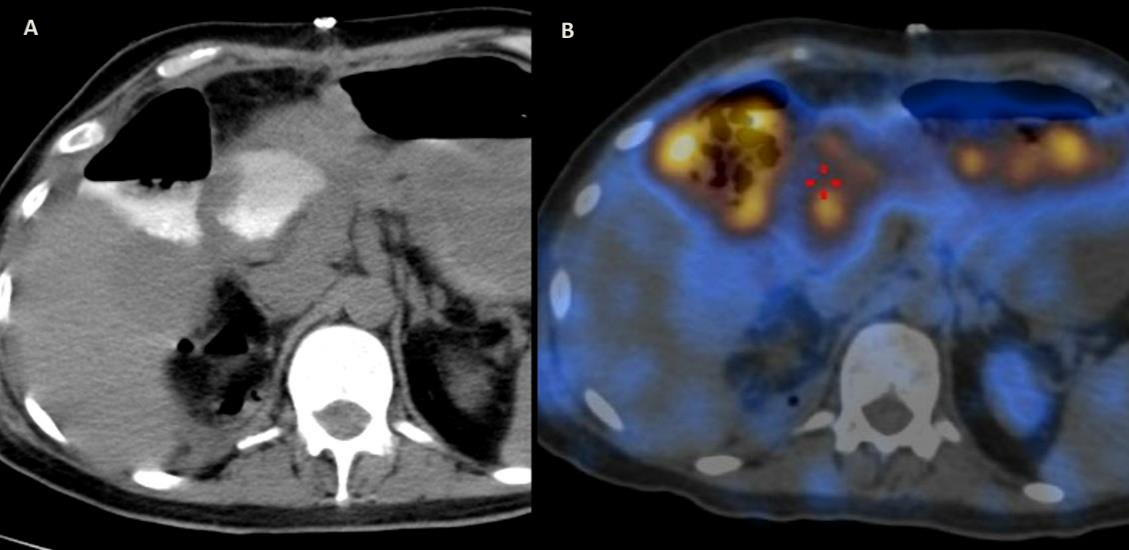
(**A**) CT image showing contrast in duodenum following contrast injection through percutaneous nephrostomy catheter (exact site of perforation was not delineable but was suspected to be through D2 segment of duodenum). (**B**) Fused image showing DTPA tracer uptake in pericolic space as well as duodenum and stomach

 Based on this suspicion, the patient was then further evaluated with serial contrast-enhanced CT abdomen. With the help of CT images (Figure 3), following contrast injection through a percutaneous nephrostomy catheter, a diagnosis of a fistulous connection between the para-duodenal collection and duodenum was made, likely through a rent in the lateral wall of the proximal third of D2 segment of the duodenum. The patient then underwent exploratory laparotomy. On duodenal C-loop dissection, a 1x1 cm opening with mucosal pouting at the D1-D2 junction of the duodenum was observed. This was plugged with omentum, and the perforation was repaired.

## Discussion

 The common diagnostic methods for identifying intraabdominal urine leak include drain creatinine studies and imaging, such as CT intravenous pyelogram. Although a retrograde pyelogram is the most accurate imaging test to evaluate the location and extent of the ureteral injury (7), a DTPA renogram can also be beneficial by delineating the site of leakage, estimating the approximate rate of leakage, and helping formulate the management strategy (8). 

 The treatment of iatrogenic ureteral injury involves different approaches depending on when it is diagnosed and how severe the injury is. The treatment options might vary from basic endoscopic management to intricate surgical reconstruction using pedicled grafts. The occurrence of urinary leakage or fistula formation following partial nephrectomy is uncommon; which can typically be controlled effectively with simple ureteric stenting. 

 However, in instances with enduring urinary leakage, laparoscopic and robotic-assisted methods have also been employed for reconstructive surgery aimed at fixing the urinary leak or fistula. In rare cases, if persistent urinary leak does not resolve using conventional methods, a completion nephrectomy may be needed. This was particularly the case in our patient who was planned for nephrectomy. It is essential to rule out any complications that may have arisen due to persistent urine leakage before proceeding with surgical interventions. 

 In our case, they wanted to know the differential function, where we raised the possibility of intestinal perforation. The etiology of intestinal perforation is multifaceted and encompasses several factors, such as trauma, instrumentation, inflammation, infection, cancer, ischemia, and obstruction. The diagnosis is typically established through clinical suspicion and traditional imaging techniques, such as X-ray and CT scans. CT scan of the abdomen and pelvis is highly efficient in detecting intestinal perforation (9). However, in early post-operative scenarios, particularly following a significant surgical procedure, clinical diagnosis of the illness becomes challenging. 

 Additionally, if the perforation is small or concealed, it may be missed using conventional radiological techniques.

 Our case may be the first case of intestinal perforation diagnosed by a DTPA renogram and subsequent SPECT/CT imaging. It shows that a renogram study, although mainly used to assess the function and drainage of kidneys, should be interpreted with caution in post-surgical cases, wherein various complications may be incidentally detected, though rarely. DTPA renogram, especially with the help of SPECT/CT, may also be used to assess the extent of urinary leak, and in rare cases may provide evidence of any perforation missed/overlooked by conventional imaging modalities.

## References

[1] Yalçın H, Özen A, Günay EC, Özaslan İA, Özer C (2011). Can 99m Tc DTPA be used in adult patients in evaluation of relative renal function measurement as the reference 99mTc DMSA method?. Mol Imaging Radionucl Ther.

[2] Morton KA, Pisani DE, Whiting JH Jr, Cheung AK, Arias JM, Valdivia S (1997). Determination of glomerular filtration rate using technetium-99m-DTPA with differing degrees of renal function. J Nucl Med Technol.

[3] Esparaz AM, Pearl JA, Herts BR, LeBlanc J, Kapoor B (2015). Iatrogenic urinary tract injuries: etiology, diagnosis, and management. Semin Intervent Radiol.

[4] Peyton CC, Hajiran A, Morgan K, Azizi M, Tang D, Chipollini J (2020). Urinary leak following partial nephrectomy: a contemporary review of 975 cases. Can J Urol.

[5] Titton RL, Gervais DA, Hahn PF, Harisinghani MG, Arellano RS, Mueller PR (2003). Urine leaks and urinomas: diagnosis and imaging-guided intervention. Radiographics.

[6] Goldwasser J, Wahdat R, Espinosa J, Lucerna A (2018). Urinoma: Prompt Diagnosis and Treatment Can Prevent Abscess Formation, Hydronephrosis, and a Progressive Loss of Renal Function. Case Rep Emerg Med..

[7] Engelsgjerd JS, LaGrange CA (2024). Ureteral Injury. [Updated 2023 Jul 4]. StatPearls [Internet].

[8] Subramanyam P, Palaniswamy SS, Tewari A, Praveen Kumar SL (2013). Iatrogenic Urinomas Identified by 99mTc DTPA Renal Scintigraphy. Trop Med Surg.

[9] Paolantonio P, Rengo M, Ferrari R, Laghi A (2016). Multidetector CT in emergency radiology: acute and generalized non-traumatic abdominal pain. Br J Radiol.

